# MORN2 regulates the morphology and energy metabolism of mitochondria and is required for male fertility in mice

**DOI:** 10.1186/s12967-024-05010-3

**Published:** 2024-03-05

**Authors:** Yining Liu, Tongtong Li, Mingze Shi, Yanling Wan, Hanzhen Li, Mingyu Zhang, Ziqi Wang, Shiyu Wang, Yue Lv, Gang Lu, Hongbin Liu, Haobo Zhang, Tao Huang

**Affiliations:** 1 Center for Reproductive Medicine, Shandong University, Jinan, 250012 Shandong China; 2 Key Laboratory of Reproductive Endocrinology of Ministry of Education, Shandong University, Jinan, 250012 Shandong China; 3 Shandong Provincial Clinical Medicine Research Center for Reproductive Health, Shandong University, Jinan, 250012 Shandong China; 4 Shandong Technology Innovation Center for Reproductive Health, Jinan, 250012 Shandong China; 5 Shandong Key Laboratory of Reproductive Medicine, Shandong Provincial Hospital affiliated to Shandong First Medical University, Jinan, 250012 Shandong China; 6 CUHK-SDU Joint Laboratory On Reproductive Genetics, School of Biomedical Sciences, The Chinese University of Hong Kong, Hong Kong, China; 7 Center for Reproductive Medicine, the Second Hospital, Cheeloo College of Medicine, Shandong University, Jinan, 250012 Shandong China

**Keywords:** Asthenospermia, Sperm motility, Mitochondrial sheath, Energy metabolism, MORN2

## Abstract

**Background:**

Mitochondria produce adenosine triphosphate through respiratory activities to power sperm differentiation and motility, and decreased mitochondrial respiratory activity can result in poor sperm motility and asthenospermia. The mitochondrial sheath is a component of the mid-piece of the sperm flagellum, and dysfunction of the sheath can reduce sperm motility and cause male infertility. The membrane occupation and recognition nexus-motif protein 2 (MORN2) is testis enriched in mice, and the MORN motif was reported to play a role in the regulation of bioelectrical signal homeostasis in cardiomyocytes.

**Methods:**

We generated *Morn2*^*–/–*^ mice using CRISPR/Cas9 and evaluated the potential functions of MORN2 in spermiogenesis through histological analysis, fertility examination, RT-PCR, CASA, immunofluorescence, TUNEL, electron microscopy analysis, mitochondrial energy metabolism analysis, etc.

**Results:**

The *Morn2*^*–/–*^ mice were infertile, and their sperm showed severe motility defects. *Morn2*^*–/–*^ sperm also had abnormal morphology characterized by bent heads, aberrant mitochondrial sheath formation, lower mitochondrial membrane potential, higher levels of reactive oxygen species, and decreased mitochondrial respiratory activity.

**Conclusions:**

Our study demonstrates that MORN2 is essential for male fertility and indicates that MORN2 functions in mitochondrial sheath formation and regulates mitochondrial respiratory activity.

**Supplementary Information:**

The online version contains supplementary material available at 10.1186/s12967-024-05010-3.

## Introduction

Spermiogenesis is a transformation phase during which round spermatids undergo acrosome formation, nuclear condensation, flagellum development, mitochondrial sheath formation, and removal of unnecessary cytoplasmic components, thus finally emerging as spermatozoa. Spermatozoa have a haploid head, a neck, and a flagellum. The flagellum is divided into three regions (the mid-piece, the principal piece, and the end piece [1]) and is the locomotive component providing the force required for delivering spermatozoa to an oocyte.

The flagellum mid-piece has a structure known as the mitochondrial sheath, which is packed tightly around the axoneme [2]. The mitochondrial sheath forms in late spermiogenesis [3], during which the sphere shaped mitochondria are recruited and undergo a series of transformation, thus finally form a double helical structure packed tightly around the axoneme [4, 5]. Although the overall number of mitochondria decreases with the loss of cytoplasm, the tightly compacted mitochondrial sheath allows the condensed mitochondria to be highly metabolically efficient [6].

Glucose is metabolized into pyruvate through glycolysis. Under aerobic condition, pyruvate could enter mitochondria and proceed tricarboxylic acid cycles (TCA cycle) [7]. Mitochondria have bilayer membranes and function in energy metabolism [8]. The metabolite of TCA cycles transport electrons in mitochondrial electron transfer chain, and finally produce adenosine triphosphate (ATP) through oxidative phosphorylation (OXPHOS) to power spermatozoa differentiation and motility [7, 8]. Previous studies have reported that spermatozoa from asthenospermia patients show aberrantly low levels of proteins that are associated with mitochondrial OXPHOS, TCA cycles, and pyruvate metabolism [9, 10], and another study showed that decreased mitochondrial respiratory activities can result in poor sperm motility and asthenospermia [11].

Besides ATP, mitochondria can also produce reactive oxidation species (ROS) through OXPHOS [12]. ROS are highly reactive oxygen-derived molecules with one or more unbound electrons [13]. At physiological concentrations, ROS are critical for the acrosomal reaction, spermatozoon-oocyte fusion, and fertilization, while excessive ROS production can result in spermatozoa damage and subsequent male infertility [12, 14]. During OXPHOS, electrons are transferred from donors to acceptors, and through this series of redox reactions the mitochondrial electron transport chain forms an electrochemical gradient that generates the mitochondrial membrane potential (MMP) [15]. A normal MMP is essential for ATP production [15], and monitoring the MMP can be used to assess mitochondrial function [16]. Low MMP has been reported to be a major cause of poor sperm motility and severe asthenospermia [17].

While conducting functional screens of testis-enriched genes in mice, we identified the membrane occupation and recognition nexus-motif protein 2 (MORN2) as a testis-enriched protein and its initial expression coincides with the first wave of meiosis [18]. A previous study predicted that MORN2 functions in the dynamic regulation of acrosome biogenesis during late spermiogenesis [18], and MORN2 has been shown to function in LC3-associated phagocytosis and in resistance to bacterial infection in planarians [19]. The MORN motif was reported to play a role in the regulation of bioelectrical signal homeostasis in cardiomyocytes [20]. However, any roles for MORN2 in spermatogenesis or reproduction remain unknown.

Aim to explore MORN2’s spermiogenesis-related functions, we generated *Morn2* knockout (*Morn2*^*–/–*^) mice using CRISPR/Cas9-mediated genome editing. Mouse *Morn2* is located on chromosome 17E3, spanning approximately 7 kb, and consists of a 669 nucleotide of open reading frame that encodes a corresponding 79 amino acid protein. Exon 4 ~ 5 was selected as target site to generate a mouse model of *Morn2*^*–/–*^*.* The *Morn2*^*–/–*^ mice were infertile and had extremely poor sperm motility. The *Morn2*^*–/–*^ sperm showed abnormal morphology, characterized by bent heads and abnormal formation of the mitochondrial sheath. We also observed that *Morn2*^*–/–*^ sperm had lower MMP, higher ROS levels, and significantly decreased ATP content, suggesting severely impaired energy metabolism. Our study demonstrates that MORN2 is essential for male fertility and indicates that it functions in mitochondrial sheath formation and in regulating mitochondrial respiratory activity. Thus, our study reveals a potential causal gene for asthenospermia and identifies a potential target for the development of male contraception technologies.

## Methods and materials

### Animals

**Fig. 1:**
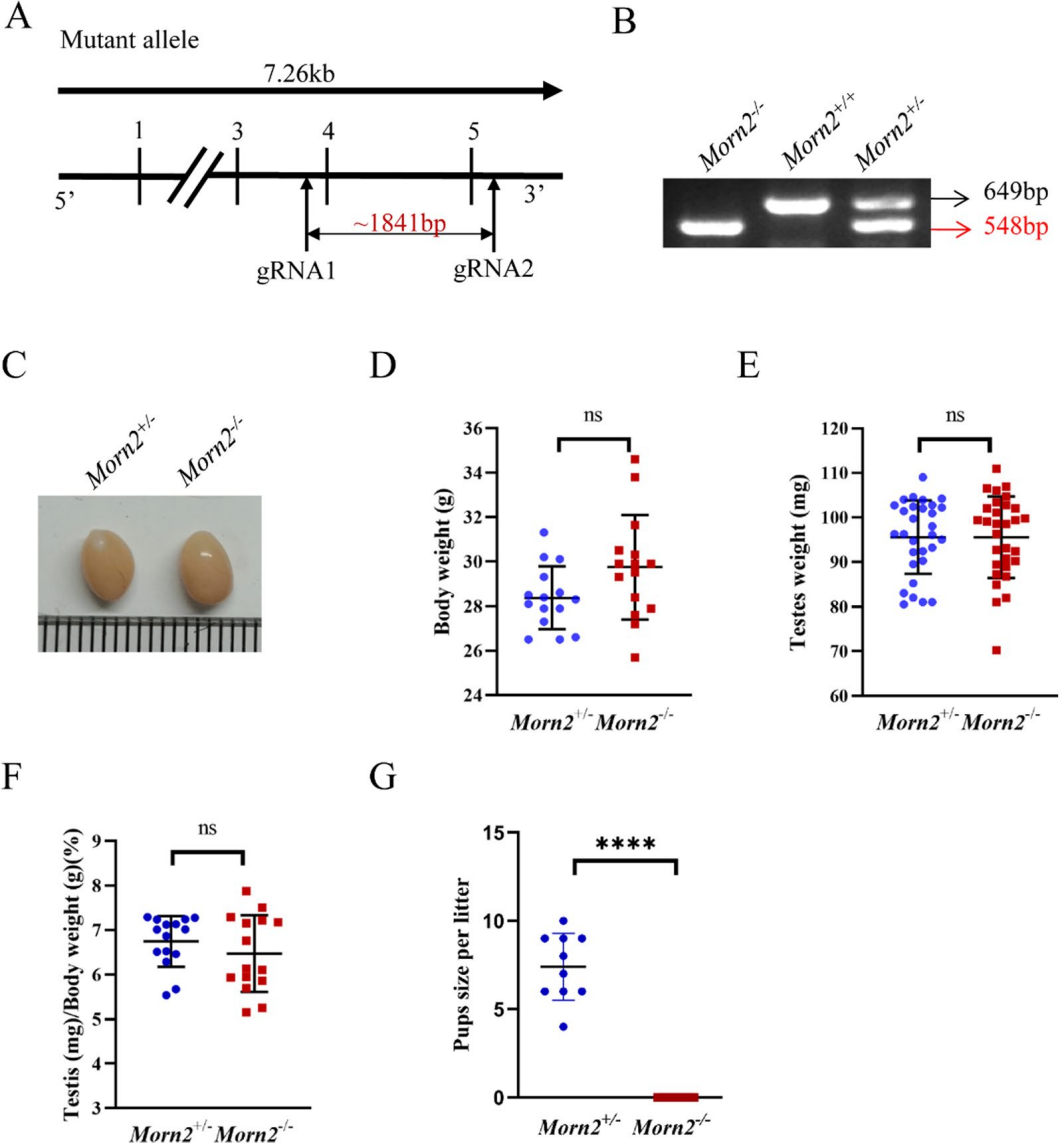
*Morn2* knockout causes male infertility. **A** Overview of the *Morn2*^*–/–*^ mouse knockout strategy. **B** Genotyping analysis using lysates prepared from the tails of *Morn2*^*–/–*^, *Morn2*^+*/*+^, and *Morn2*^+*/–*^ mice showed successful knockout of *Morn2*. **C** Representative photographs of testes of *Morn2*^+*/–*^ and *Morn2*^*–/–*^ mice. **D** The body weight of adult *Morn2*^+*/–*^ and *Morn2*^*–/–*^ mice. Data are shown as the mean ± SEM of 15 independent experiments, each data point represents the body weight of an individual, and ns = P > 0.05 by Student’s t-test. **E** The testes weight of adult *Morn2*^+*/–*^ and *Morn2*^*–/–*^ mice. Data are shown as the mean ± SEM of 15 independent experiments, each data point represents the testes weight of an individual, and ns = P > 0.05 by Student’s t-test. **F** Quantification of the testes weight/body weight ratio in *Morn2*^+*/–*^ and *Morn2*^*–/–*^ mice. Data are shown as the mean ± SEM of 15 independent experiments, each data point represents the testis-to-body weight ratio of an individual, and ns = P > 0.05 by Student’s t-test. **G** Number of pups per litter from *Morn2*^+*/–*^ and *Morn2*^*–/–*^ male mice. In *Morn2*^+*/–*^mice, the mean number of pups per litter from 10 litters was 7.40 ± 1.90. In *Morn2*^*–/–*^ mice, no pups were born. Data are shown as the mean ± SEM of 3 independent experiments, each data point represents the number of pups per litter of an individual, and **** = P < 0.0001 by Student’s t-test

The Morn2 gene (Transcript: ENSMUST00000061703) is located on mouse chromosome 17, containing 5 exons. As the ATG start codon was identified in exon4 and the TAG stop codon was identified in exon 5, the exon 4 ~ 5 was selected as target site to generate a mouse model of *Morn2*^*–/–*^ in C57BL/6 background via CRISPR/Cas9-mediated genome editing system (Cyagen Biosciences, USA). Briefly, Cas9 and gRNA was co-injected into fertilized eggs of C57BL/6 mice. Fertilized eggs were transplanted to generate positive F0 mice (*Morn2*^+*/–*^) which had a targeted line with a 1841 bp base deletion (Fig. 1A). The pups were genotyped by polymerase chain reaction (PCR) following with DNA sequencing analysis. The stable F1 generation mouse model was obtained by mating positive F0 generation mice with C57BL/6 mice. The *Morn2*^+*/–*^ male mice (F1) were housed with *Morn2*^+*/–*^ female mice (F1) to generate *Morn2*^*–/–*^ mice. All mice were housed in the specific pathogen-free (SPF) animal facility. All the animal experiments were performed according to approved Animal Use Committee of the School of Medicine protocols, Shandong University.

### Real-time PCR

Total RNA was isolated from multiple adult tissues (including liver, spleen, lung, kidney, uterus, ovary and testis) and 8–56-d-old mice testes of wild-type C57BL/6 mice via FastPure Cell/Tissue Total RNA Isolation Kit V2 (Vazyme, RC112-01) following manufacturer's instructions. All the cDNAs were prepared using the HiScript Q RT SuperMix for qPCR (Vazyma, R323-01) following the manufacturer’s instructions. The RT-PCR experiments were initiated with denaturation at 95 ℃ for 5 min, followed by 35 cycles of denaturation at 95 ℃ for 30 s, annealing at 60 ℃ for 30 s and extension at 72 ℃ for 1 min, then finally extension at 72 ℃ for 10 min using a T100 Thermal Cycler (Bio-Rad). The sequences of primers used for *Morn2* were Forward:5-CGTCTCCATCAACTTCGTC-3 and Reverse:5-CTGTCCCGTTTCTCTCACA-3. *Gapdh* was amplified as a housekeeping gene with the primers Forward:5-GCCTTCTCCATGGTGGTGAA-3 and Reverse:5-GCACAGTCAAGGCCGAGAAT-3. The RT-PCR experiments was replicated using distinct biological samples for at least three time.

### Tissue collection and histological analysis

Testes and epididymides of mice were dissected immediately after euthanasia, fixed in 4% (mass/vol) paraformaldehyde (PFA) (Solarbio, Beijing, China, P1110) or Bouin’s solution (Sigma, HT10132) for up to 24 h, dehydrated in into 70% (vol/vol) ethanol, and embedded in paraffin. The tissues were sectioned at 5 μm and mounted on glass slides. After deparaffinized and rehydrated, the sections were sequentially stained with hematoxylin(H), hematoxylin–eosin (H&E), or PAS for histological analysis. The histological analysis was replicated using distinct biological samples for at least three time.

### Sperm collection and immunofluorescence in spermatozoa

The caudal epididymides were dissected immediately from *Morn2*^+*/–*^ and *Morn2*^*–/–*^ mice after euthanasia. Spermatozoa were squeezed out from the caudal epididymides and released in 1 ml phosphate buffered saline (PBS) for 20 min at 37 ℃ under a 5% CO_2_ atmosphere for spermatozoa releasing. 20 μl sample was spread on glass slides, air-dried, and stored at − 80 °C for further morphological analysis and immunofluorescence experiments. After being fixed in 4% PFA for 15 min, washed 3 times using PBS and rehydrated, the slides were stained with hematoxylin and eosin (HE) for histological analysis.

For immunofluorescence, the slides were fixed in 4% PFA for 15 min at room temperature, and were washed 3 times in 1 × PBS. Subsequently, the slides were blocked using 5% BSA in a humidified chamber for at least 30 min at room temperature. The slides were then incubated with primary antibodies (DNALI1, SPEF2, SPAG6) in a humidified chamber at 4 ℃ overnight. After that, the slides were washed 3 times using PBS and incubated with secondary antibodies at room temperature for 1 h. Then the slides were washed 3 times using PBS again and were finally stained by mounting medium with DAPI-Aqueous (Abcam, ab104139) and images were acquired on an SP8 microscope (Leica). The primary and secondary antibodies used and their dilution are listed in Additional file 1: Table S1. Each immunofluorescence experiment was replicated using distinct biological samples for at least three time.

Given that some antibodies (PNA, mito tracker, TOMM20,) used in this study are directly conjugated with fluorescence, the slides can be incubated with these antibodies for one hour at room temperature after blocking. Then the slides were washed 3 times using PBS again and were finally stained by mounting medium with DAPI-Aqueous (Abcam, ab104139) and images were acquired on an SP8 microscope (Leica).

### Immunofluorescence in testes

The paraffin-embedded sections were prepared as previously described. The sections were deparaffinized, rehydrated and followed by antigen retrieval with 10 mM sodium citrate buffer (pH 6.0) (Proteintech, PR30001) in boiling water for 15 min, cooling down at room temperature, and then treated with PBS containing 0.1% Triton X-100. The subsequent washing, blocking and incubating were consistent with the protocol described previously for sperm spread slides. Subsequently, the slides were washed 3 times in 1 × PBS and then blocked using 5% BSA in a humidified chamber for at least 30 min at room temperature. The slides were then incubated with primary antibodies (GOPC, GM130) in a humidified chamber at 4 ℃ overnight. After that, the slides were washed 3 times using PBS and incubated with secondary antibodies at room temperature for 1 h. Then the slides were washed 3 times using PBS again and were finally stained by mounting medium with DAPI-Aqueous (Abcam, ab104139) and images were acquired on an SP8 microscope (Leica). The primary and secondary antibodies used and their dilution are listed in Additional file 1: Table S1. Each immunofluorescence experiment was replicated using distinct biological samples for at least three time.

### TUNEL assay

The protocol of TUNEL staining was followed by the manufacturer's instructions (keyGEN BioTECH, #KGA7072). Briefly, the paraffin-embedded sections were prepared as previously described. The sections were deparaffinized, rehydrated and followed by antigen retrieval with 10 mM sodium citrate buffer (pH 6.0) (Proteintech, PR30001) in boiling water for 15 min, cooling down at room temperature, and then treated with PBS containing 0.1% Triton X-100. After washed 3 times in 1 × PBS, the sections were incubated with TdT enzyme reaction buffer (45 μl Equilibration Buffer + 1.0 μl biotin-11-dUTP + 4.0 μl TdT Enzyme per sample) for 60 min at 37 ℃. Subsequently, each section was washed 3 times with 1 × PBS, and then labeled with 50 μl Streptavidin-Fluorescein labeling buffer (45 μl Labeling Buffer + 5.0 μl Streptavidin-Fluorescein) for 30 min at 37 ℃. Finally, the sections were washed with 1 × PBS for 3 times and stained by mounting medium with DAPI-Aqueous (Abcam, ab104139). The TUNEL assay was replicated using distinct biological samples for at least three time.

### Sperm motility analysis using CASA

The sperm samples from at least three *Morn2*^+*/–*^ and *Morn2*^*–/–*^ mice were performed sperm motility assessment analysis using an Olympus B × 51 microscope (Olympus, Tokyo, Japan) through a 20 × phase objective. 10 μl suspended sperm liquid were taken from each sample and placed into 80 μm deep glass cell chambers (Hamilton Thorne, 80-micron 2X-CEI), and then captured by CCD camera. Via the Animal Motility system (Hamilton Thorne, Beverly, MA, USA), more than 200 spermatozoa were performed by computer-associated semen analysis (CASA) to evaluate motility parameters, including the total motile spermatozoa, progressive spermatozoa, average path velocity (VAP), progressive velocity (VSL), the track speed (VCL). The semen analysis was replicated using distinct biological samples at least three times.

### In vitro/vivo fertilization

Oocytes collected from superovulating WT females (at age of 5 weeks) were placed in mHTF medium covered with paraffin oil. Spermatozoa were collected from the cauda epididymides of *Morn2*^+*/–*^ and *Morn2*^*–/–*^ mice (age of 12–16 weeks) and incubated in a TYH drop for 30 min at 37 ℃ under 5% CO_2_ atmosphere. Then the eggs were then cultured with spermatozoa for 6 h, and the fertilization rate was determined at 24 h by counting the number of two-cell embryos.

For in vivo fertilization, individual males (WT and KO) were housed with superovulating WT females, and copulatory plugs were detected at 9 am on the next day. 36 h after mating, the fallopian tube of the female mice was dissected, and the contents of the ampullae tubae uterinae were flushed out with M2 medium (Sigma), then the fertilization rate was determined by counting the number of two-cell embryos. The fertilization assay was replicated using distinct biological samples for at least three time.

### Transmission electron microscopy analysis (TEM)

The caudal epididymides were dissected immediately from *Morn2*^+*/–*^ and *Morn2*^*–/–*^ mice (n = 3) after euthanasia, spermatozoa were squeezed out from the caudal epididymides. After centrifugation and washing, the sediments were suspended and fixed overnight at 4 ℃ in 2.5% glutaraldehyde, sheltered from light. Then, the samples were immersed in 1% OsO4 for 1 h at 4 ℃ sheltered from light, and washed with distilled water for 4 times. Subsequently, the samples were dehydrated through a graded acetone series and embedded in resin ((Low Viscosity Embedding Media Spurr’s Kit, EMS, 14300) for staining. Finally, haven been cut on an ultramicrotome, the sample ultrathin sections were double stained with uranyl acetate and lead citrate, and been imaged and analyzed using a transmission electron microscope (TEM) (JEM-1400, JEOL, Tokyo, Japan). The TEM analysis was replicated using distinct biological samples for at least three time.

### Scanning electron microscopy analysis (SEM)

The methods of sampling, fixation, and washing of samples were consistent with those mentioned in TEM above. After washing, the samples were dehydrated through cold 50%, 70%, 95%, and 100% ethanol. Sequentially, the samples were dried at critical point of 55 ℃ using Tousimis Autosamdri-810 Critical Point Dryer, mounted onto specimen stubs, and coated with palladium. Finally, the samples were observed and analyzed with a FEI Quanta ESEM 200 SEM. The SEM analysis was replicated using distinct biological samples for at least three time.

### Evaluation of mitochondrial membrane potential

We have selected JC-1 as the probe for detecting mitochondrial membrane potential. When the mitochondrial membrane potential is high, JC-1 aggregates in the mitochondrial matrix, forming polymers (J-aggregates) that emit red fluorescence. Conversely, at lower membrane potentials, JC-1 cannot aggregate in the matrix and exists as monomers, emitting green fluorescence. The relative ratio of red to green fluorescence was used to measure the proportion of mitochondrial depolarization. The protocol was followed by the manufacturer’s instructions (Beyotime, China, C2006). Briefly, the caudal epididymides were dissected immediately from *Morn2*^+*/–*^ and *Morn2*^*–/–*^ mice after euthanasia, spermatozoa were squeezed out from the caudal epididymides. Then the spermatozoa were incubated in the pre-configured 0.5 ml 1X JC-1 working solution at 37 ℃ for 20 min. After centrifugation and washing with 1 ml JC-1 staining buffer (1X) for 3 times, the sediments was suspended with 200 μl JC-1 staining buffer (1X) and images were acquired immediately on an SP8 microscope (Leica). The quantification of fluorescence values was performed with the use of ImageJ. The MMP analysis was replicated using distinct biological samples at least three times.

### Evaluation of reactive oxidation species

We utilized the Reactive Oxygen Species Assay Kit (also known as ROS Assay Kit) (Beyotime, China, S0033) to assess the levels of ROS in sperm. The ROS assay kit employs the fluorescent probe DCFH-DA for the detection of ROS. DCFH-DA itself is non-fluorescent, and upon entering the cell, it undergoes hydrolysis by esterases to generate DCFH. ROS within the cell can oxidize non-fluorescent DCFH, resulting in the production of fluorescent DCF. Monitoring the fluorescence of DCF allows us to determine the level of ROS inside the cells. The protocol was followed by the manufacturer's instructions. Briefly, the caudal epididymides were dissected immediately from *Morn2*^+*/–*^ and *Morn2*^*–/–*^ mice after euthanasia, spermatozoa were squeezed out from the caudal epididymides. Then the spermatozoa were incubated in the pre-configured 0.5 ml DCFH-DA solution (10 μM/L) at 37 ℃ for 20 min. After centrifugation and washing with PBS for 3 times, the sediments was suspended with PBS and images were acquired immediately on an SP8 microscope (Leica). The quantification of fluorescence values was performed with the use of ImageJ. The ROS analysis was replicated using distinct biological samples at least three times. For more accurate quantification of ROS levels in spermatozoa, we also quantified the fluorescence intensity using a fluorescence luminometer. The stained samples were transferred into a black 96-well plate. Each detection hole on the black 96-well plate was added 100 μl sample, and 3 replicates were set for each sample. Fluorescence intensity was measured immediately using a luminometer. To minimize variability between different experimental batches, data analysis was performed using the average of three replicates per sample. The results for *Morn2*^+*/*+^ mice were represented as 1, and those for *Morn2*^*−/−*^ mice were expressed as the ratio of *Morn2*^*−/−*^ to *Morn2*^+*/*+^. The fluorescence luminometer analysis was replicated using distinct biological samples for at least three time.

### Measurement of ATP concentration in spermatozoa

The protocol was followed by the manufacturer's instructions (Beyotime, China, S0026). Briefly, the caudal epididymides were dissected immediately from *Morn2*^+*/–*^ and *Morn2*^*–/–*^ mice after euthanasia, spermatozoa were squeezed out from the caudal epididymides. Then the spermatozoa were incubated in the pre-configured 200 μl ATP test lysate, the lysate was Vortexed gently for complete reaction. After centrifugation, the supernatant was taken for subsequent determination (sample lysate was performed at 4 °C or on ice). The ATP standard solution was diluted with the ATP test lysate into a graded series of 0.01, 0.03, 0.1, 0.3, 1, 3 and 10 µM. Each detection hole on the black 96-well plate was added 100 μl of pre-prepared ATP detection solution. After 3–5 min waiting for the consumption of background ATP, 20 μl samples or standard products were added into the detection hole and were mixed quickly with a micropipette. 3 replicates were set for each sample. After 3 min interval, the RLU value was measured by luminometer. The ATP concentration measurement analysis was replicated using distinct biological samples for at least three times.

### Statistical Analysis

All of the experiments were repeated at least three times, and the results were presented as the mean ± SEM. The statistical significance of the differences between the mean values for the different genotypes was measured by the student’s t-test with a paired, two-tailed distribution. The data were considered significant for P < 0.05.

## Results

### *Morn2* is an evolutionarily conserved and testis-enriched protein

In the Ensembl database (http://asia.ensembl.org/index.html) we found that MORN-motif protein 2 is highly evolutionarily conserved in several species, including humans, chimpanzees, dogs, cows, rats, etc. Consistent with the expression profile in the Ensembl database (http://asia.ensembl.org/index.html), our RT-PCR analysis of multiple organs, including the liver, spleen, lung, kidney, uterus, ovary, and testis, from wild type (WT) mice showed that *Morn2* was expressed specifically in the testes (Additional file 1: Fig. S1A). We also performed a developmental time series RT-PCR analysis of testes and found that *Morn2* was detected in the testicular tissue of mice at the postnatal day 8 (PD8) stage (Additional file 1: Fig. S1B), and persisted throughout subsequent developmental stages. By adulthood (PD56), the expression of *Morn2* in the testicular tissue of mice was maintained at a higher level.

### *Morn2* knockout causes male infertility

To characterize the potential functions of *Morn2* during spermatogenesis, we engaged Cyagen Biosciences for the generation of *Morn2*^*–/–*^ mice using CRISPR/Cas9. We selected the region comprising exon 4 to exon 5 as the target site for an 1841 bp deletion edit (Fig. 1A). Genomic DNA sequencing of the founder animals confirmed the desired genome edit (Fig. 1B). Heterozygous genome-edited mice had normal morphology and could produce healthy offspring.

There were no obvious gross phenotypes in the *Morn2*^*–/–*^ mice, and compared to the *Morn2*^+*/–*^ mice, the *Morn2*^*–/–*^ mice showed no significant differences in testis size, body weight, testis weight, or the ratio of testes weight to body weight (Fig. 1C–F). We examined the fertility of male *Morn2*^*–/–*^ mice, and individual males (*Morn2*^+*/–*^ and *Morn2*^*–/–*^) were housed with three WT females for 4 months, and copulatory plugs were detected at 9 AM on the next day of mating with both *Morn2*^+*/–*^ and *Morn2*^*–/–*^ males. No pups were born from *Morn2*^*–/–*^ male mice (Fig. 1G), whereas the *Morn2*^+*/–*^ males successfully sired offspring with an average pup number of 7.40 ± 1.90 (n = 3) per litter, suggesting MORN2 is essential for male fertility.

### *Morn2*^*–/–*^ spermatozoa exhibit bent heads

**Fig. 2:**
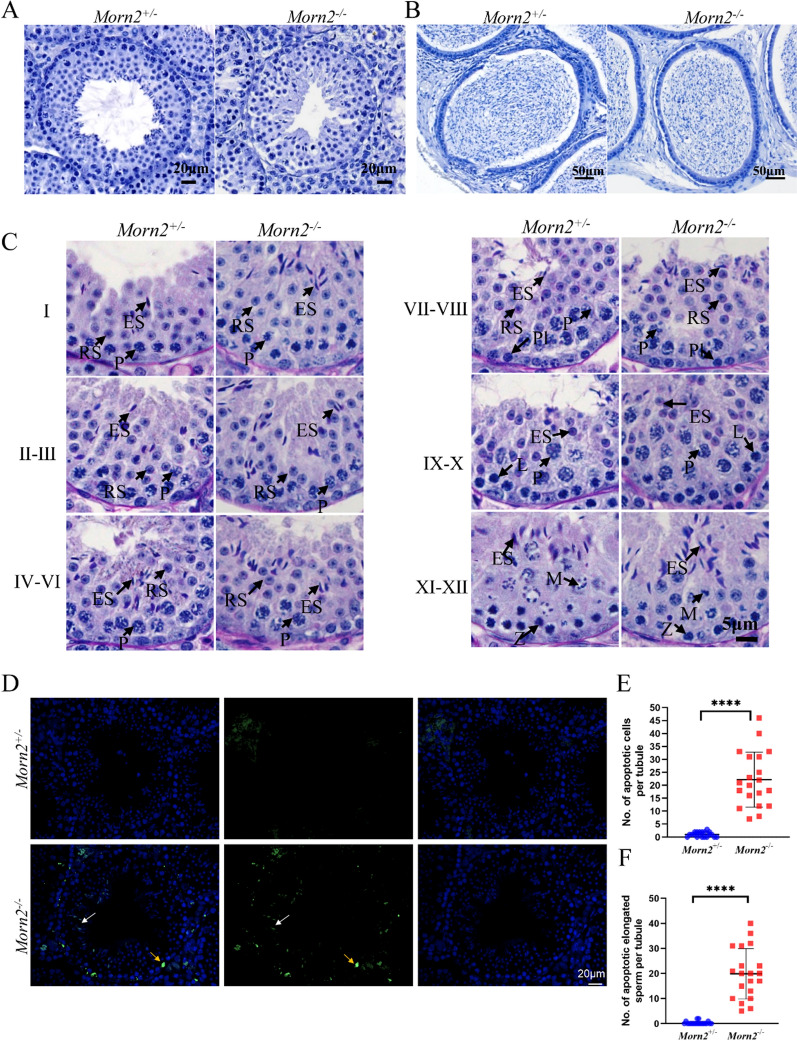
The number of apoptotic cells increased in *Morn2*^*–/–*^ mice. **A** Hematoxylin staining of testes in *Morn2*^+*/–*^ and *Morn2*^*–/–*^ mice. The test was replicated three times using distinct biological samples. The scale bar is 20 μm. **B** Hematoxylin staining of epididymides in *Morn2*^+*/–*^ and *Morn2*^*–/–*^ mice. The test was replicated three times using distinct biological samples. The scale bar is 50 μm. **C** PAS staining of seminiferous tubules from different stages in *Morn2*^+*/–*^ and *Morn2*^*–/–*^ mice. (Pl, preleptotene spermatocytes; L, leptotene spermatocytes; Z, zygotene spermatocytes; P, pachytene spermatocytes; RS, round sperm; ES, elongated sperm; M, metaphase of meiosis. The test was replicated three times using distinct biological samples. The scale bar is 5 μm.). **D** TUNEL staining of seminiferous tubules showed that there were more apoptotic cells in *Morn2*^*–/–*^ mice compared with *Morn2*^+*/–*^ mice, and most of the apoptotic cells were round and elongated sperm. The white arrow indicates an apoptotic elongated sperm, and the yellow arrow indicates an apoptotic spermatocyte. The test was replicated three times using distinct biological samples. The scale bar is 20 μm. **E** The number of apoptotic cells per seminiferous tubule in *Morn2*^+*/–*^ mice (1.05 ± 0.92) and *Morn2*^*–/–*^ mice (22.20 ± 10.38). The data are shown as the mean ± SEM of three independent experiments using distinct biological samples. Each data point represents the number of apoptotic cells per seminiferous tubule, **** = P < 0.0001 by Student’s t-tests. **F** The number of apoptotic elongated sperm per seminiferous tubule in *Morn2*^+*/–*^ mice (0.30 ± 0.64) and *Morn2*^*–/–*^ mice (19.90 ± 9.79). The data are shown as the mean ± SEM of three independent experiments using distinct biological samples. Each data point represents the number of apoptotic elongated sperm per seminiferous tubule, **** = P < 0.0001 by Student’s t-tests

Hematoxylin staining of testes and epididymides sections from *Morn2*^*–/–*^ mice showed no obvious abnormalities in the morphology or development of the seminiferous tubules, nor were there any differences in the spermatozoa number in the epididymides between the *Morn2*^+*/–*^ and *Morn2*^*–/–*^ mice (Fig. 2A, B). Periodic Acid-Schiff (PAS) staining of testes sections showed all of the expected seminiferous epithelium components and developmental stages in both *Morn2*^+*/–*^ and *Morn2*^*–/–*^ mice (Fig. 2C). Together, these results indicated that the absence of *Morn2* had no significant effect on prophase in spermiogenesis.

Given a previous study reporting that *Morn2* might be associated with dynamic regulation of acrosome biogenesis during late spermiogenesis[18], we examined acrosome biogenesis with PNA (marker of acrosome) in spermatozoa spreads using immunofluorescence staining and found no abnormalities between *Morn2*^+*/–*^ and *Morn2*^*–/–*^ mice (Additional file 1: Fig. S2A). In light of previous studies confirming that the dynamic trafficking of Golgi-derived vesicles is also involved in acrosome biogenesis[21], we performed multiple immunofluorescence staining experiments (with GOPC/GM130 as the marker for the Golgi apparatus), but we found no differences between the testes of *Morn2*^+*/–*^ and *Morn2*^*–/–*^ mice (Additional file 1: Fig. S2B, C), indicating that acrosome genesis occurs normally in *Morn2*^*–/–*^ testes.

TUNEL staining of testicular sections showed that *Morn2*^*–/–*^ mice had an increased number of apoptotic cells in their seminiferous tubules compared to *Morn2*^+*/–*^ mice (Fig. 2D, E), and these cells were concentrated in the elongated spermatozoa (Fig. 2F), suggesting that *Morn2* may affect late spermatogenesis.

**Fig. 3:**
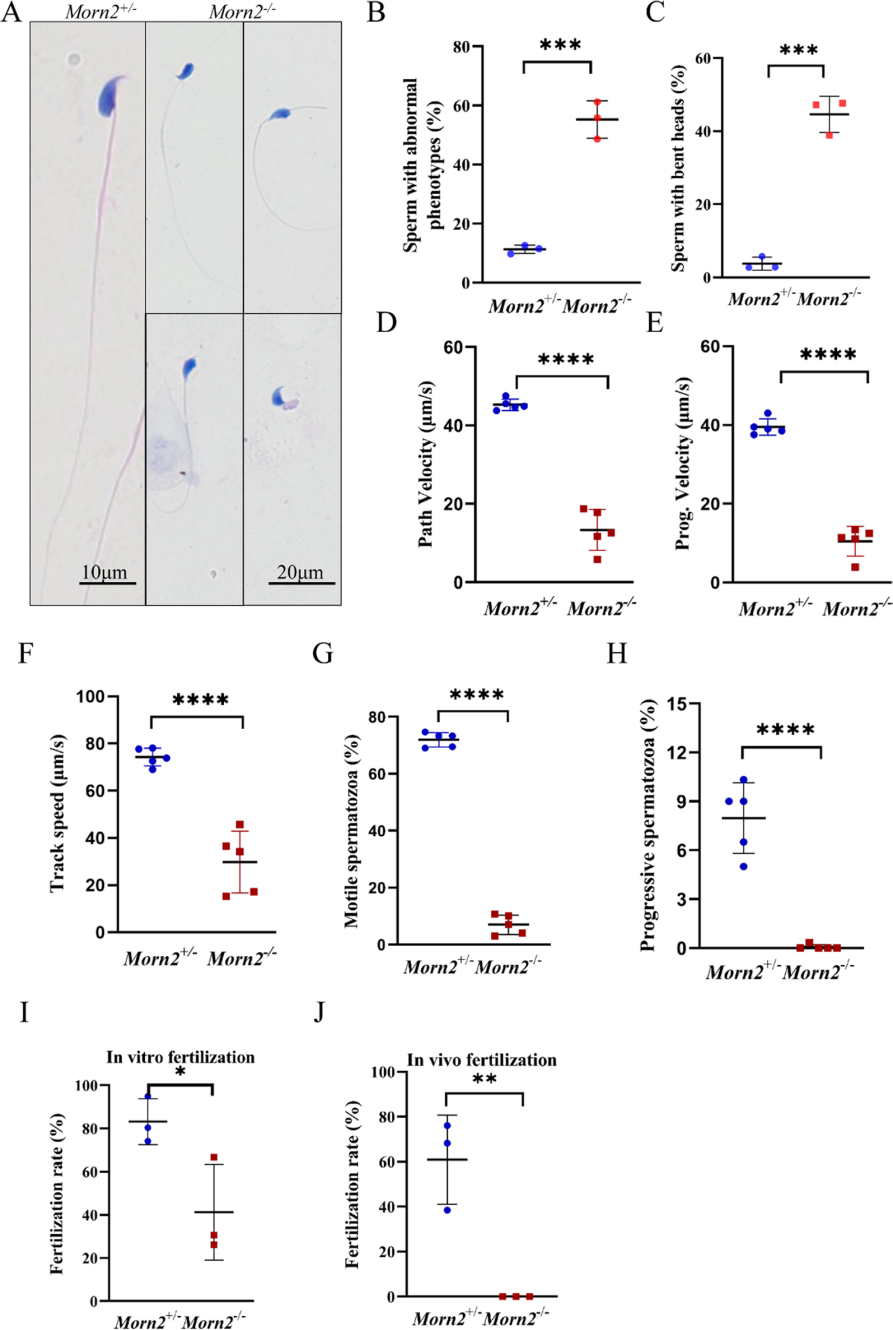
*Morn2*^*–/–*^ sperm exhibit bent heads and significantly decreased motility. **A** Hematoxylin and eosin staining of epididymal sperm smears in *Morn2*^+*/–*^ and *Morn2*^*–/–*^ mice. The test was replicated three times using distinct biological samples. The scale bar is 20 μm. **B** The percentage of epididymal sperm with abnormal phenotypes in *Morn2*^+*/–*^ (11.28 ± 1.41%) and *Morn2*^*–/–*^ (55.21 ± 6.32%) mice. At least 200 spermatozoa were counted in each sample. The data are shown as the mean ± SEM of three independent experiments using distinct biological samples. Each data point represents the percentage of sperm with abnormal phenotypes per sample, *** = P < 0.001 by Student’s t-tests. **C** The percentage of epididymal sperm with bent heads in *Morn2*^+*/–*^ (3.75 ± 1.78%) and *Morn2*^*–/–*^ (44.59 ± 4.94%) mice. At least 200 spermatozoa were counted in each sample. The data are shown as the mean ± SEM of three independent experiments using distinct biological samples. Each data point represents the percentage of sperm with bent heads per sample, *** = P < 0.001 by Student’s t-tests. **D** The VAP of spermatozoa in *Morn2*^+*/–*^ (45.26 ± 1.42 μm/s) and *Morn2*^*–/–*^ (13.34 ± 5.21 μm/s) mice (n = 3). At least 200 spermatozoa were counted in each sample. The data are shown as the mean ± SEM of three independent experiments using distinct biological samples. Each data point represents the VAP of spermatozoa in an individual, **** = P < 0.0001 by Student’s t-tests. **E** The VSL of spermatozoa in *Morn2*^+*/–*^ (39.52 ± 2.08 μm/s) and *Morn2*^*–/–*^ (10.49 ± 3.78 μm/s) mice (n = 3). At least 200 spermatozoa were counted in each sample. The data are shown as the mean ± SEM of three independent experiments using distinct biological samples. Each data point represents the VSL of spermatozoa in an individual, **** = P < 0.0001 by Student’s t-tests. **F** The VCL of spermatozoa in *Morn2*^+*/–*^ (75.26 ± 3.76 μm/s) and *Morn2*^*–/–*^ (29.81 ± 13.11 μm/s) mice (n = 3). At least 200 spermatozoa were counted in each sample. The data are shown as the mean ± SEM of three independent experiments using distinct biological samples. Each data point represents the VCL of spermatozoa in an individual, **** = P < 0.0001 by Student’s t-tests. **G** The percentage of motile spermatozoa in *Morn2*^+*/–*^ (71.97 ± 2.55%) and *Morn2*^*–/–*^ (6.93 ± 3.44%) mice. At least 200 spermatozoa were counted in each sample. The data are shown as the mean ± SEM of three independent experiments using distinct biological samples. Each data point represents the percentage of motile spermatozoa in an individual, **** = P < 0.0001 by Student’s t-tests. **H** The percentage of progressive motile spermatozoa in *Morn2*^+*/–*^ (7.97 ± 2.16%) and *Morn2*^*–/–*^ (0.07 ± 0.15%) mice. At least 200 spermatozoa were counted in each sample. The data are shown as the mean ± SEM of three independent experiments using distinct biological samples. Each data point represents the percentage of progressive motile spermatozoa in an individual, **** = P < 0.0001 by Student’s t-tests. **I** The IVF rate with cumulus-intact oocytes of *Morn2*^+*/–*^ (83.14 ± 10.63%) and *Morn2*^*–/–*^ (41.18 ± 22.19%) mice. The experiment involved 3 *Morn2*^+*/–*^ or *Morn2*^+*/*+^ male mice, 3 *Morn2*^*–/–*^ male mice, and 30 WT female mice, with a total of 446 oocytes included in the analysis. The data are shown as the mean ± SEM of three independent experiments. Each data point represents the fertilization rate of a male individual, *P < 0.05 by Student’s t-tests. **J** The in vivo fertilization rate of *Morn2*^+*/–*^ (60.88 ± 19.81%) and *Morn2*^*–/–*^ (0%) mice. The experiment involved 3 *Morn2*^+*/–*^ or *Morn2*^+*/*+^ male mice, 3 *Morn2*^*–/–*^ male mice, and 12 WT female mice. The data are shown as the mean ± SEM of three independent experiments. Each data point represents the fertilization rate of a male individual, ** = P < 0.01 by Student’s t-tests

Subsequent hematoxylin and eosin staining of sperm spreads revealed abnormal phenotypes in *Morn2*^*–/–*^ mouse samples, including bent heads (the aberrant sperm were bent from the neck, and their heads were attached to the flagellum), bent flagellums, and coiled tails (Fig. 3A). The proportion of deformed spermatozoa in *Morn2*^*–/–*^ was about 55.21 ± 6.32%, while that in *Morn2*^+*/–*^ was only 11.28 ± 1.41% (Fig. 3B). The proportion of spermatozoa with bent heads in *Morn2*^*–/–*^ mice was about 44.59 ± 4.94%, while that in *Morn2*^+*/–*^ was only 3.75 ± 1.78% (Fig. 3C), but there were no significant differences in the proportions of spermatozoa with bent flagellums or coiled tails between *Morn2*^+*/–*^ and *Morn2*^*–/–*^ mice (Additional file 1: Fig. S2D, E).

We then performed hematoxylin and eosin staining of spreads with sperm from caput, corpora, and caudal epididymides respectively. Segmented counting results in the caput, corpus, and cauda regions of the epididymis revealed that the quantity of abnormal sperm in *Morn2*^*–/–*^ mice was significantly higher than that in the *Morn2*^+*/–*^ mice of the corresponding period (Additional file 1: Fig. S2F), which indicated that the abnormal morphology of spermatozoa appeared before entering the epididymis. Thus, it suggested that MORN2 might play a role in the pre-epididymis stage.

### *Morn2*^*–/–*^ spermatozoa have significantly decreased motility

We next examined whether the morphological abnormalities in *Morn2*^*–/–*^ sperm affect motility. Computer-associated spermatozoa analysis (CASA) revealed significant decreases in the average path velocity (VAP), progressive velocity (VSL), and track speed (VCL) of *Morn2*^*–/–*^ sperm compared to *Morn2*^+*/–*^ sperm (Fig. 3D–F). In addition, the proportion of motile spermatozoa was only 6.93% in *Morn2*^*–/–*^ mice and the proportion of progressive spermatozoa was only 0.07%, while in *Morn2*^+*/–*^ mice these two values were 71.97% and 7.97%, respectively (Fig. 3G, H). These results indicated that the motility of *Morn2*^*–/–*^ spermatozoa was significantly decreased.

### *Morn2*^*–/–*^ spermatozoa could complete fertilization in vitro

To examine whether spermatozoa from *Morn2*^*–/–*^ mice undergo capacitation and the acrosome reaction, we performed in vitro fertilization (IVF) analysis with male *Morn2*^*–/–*^ mice and WT females. We observed successful fertilization in *Morn2*^*–/–*^ males when IVF was performed with cumulus-intact oocytes (Fig. 3I), although the fertilization rate of *Morn2*^*–/–*^ mice was decreased compared to *Morn2*^+*/–*^ mice. This finding indicated that spermatozoa from *Morn2*^*–/–*^ mice were able to penetrate the zona pellucida (ZP) and fuse with oocytes. Subsequently, we performed in vivo fertilization experiments by housing *Morn2*^+*/–*^ and *Morn2*^*–/–*^ male mice with superovulating WT females, and we measured the fertilization rate by counting the number of two-cell embryos flushed from the ampullae tubae uterinae 36 h after mating. Despite the observation of copulatory plugs, no two-cell embryos were detected in females mated with *Morn2*^*–/–*^ males (Fig. 3J), indicating that *Morn2*^*–/–*^ sperm could not complete fertilization in vivo.

### *Morn2*^*–/–*^ sperm show abnormal mitochondrial morphology

**Fig. 4:**
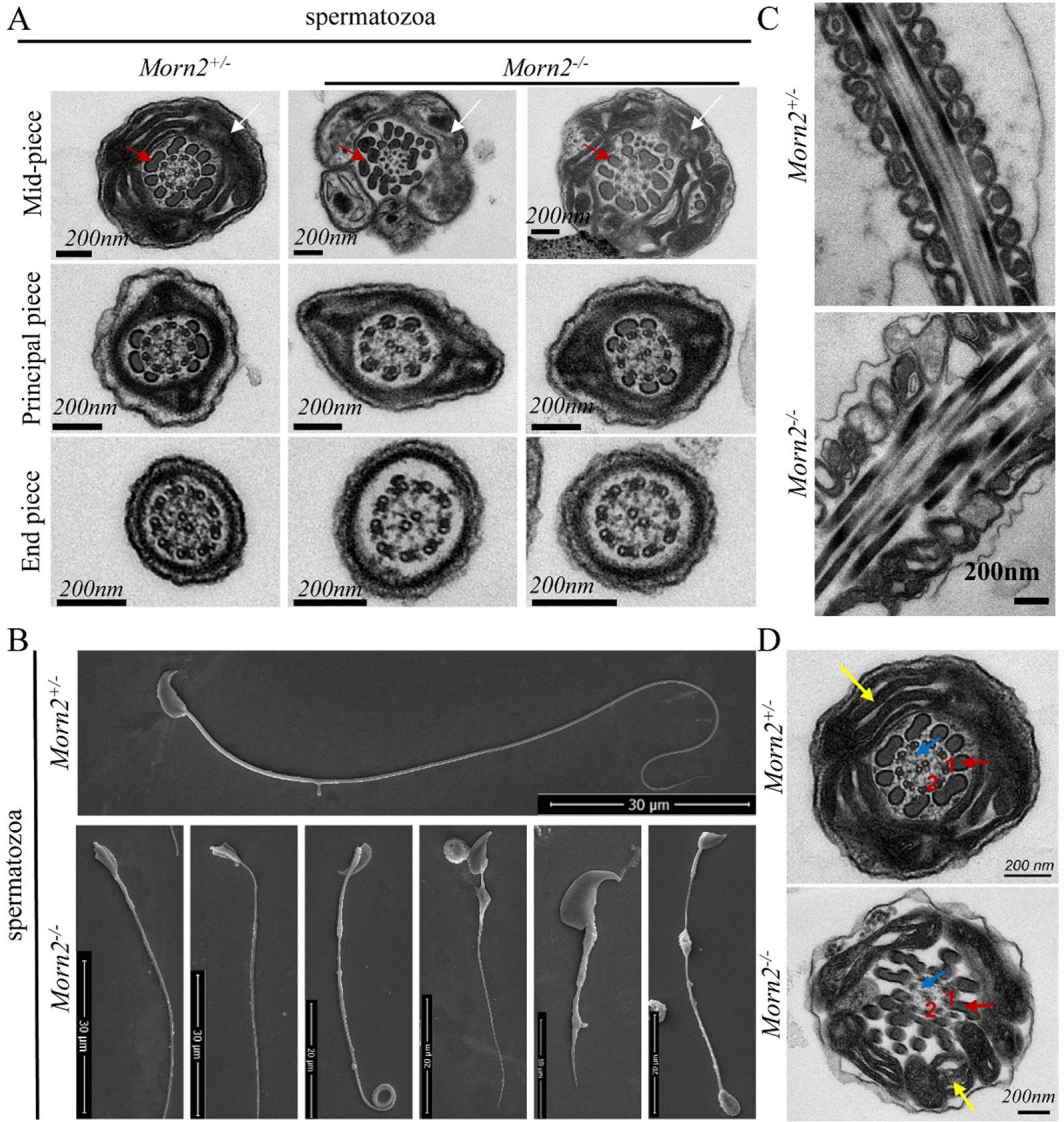
*Morn2* knockout causes abnormal mitochondrial morphology in sperm. **A** TEM analysis of spermatozoa from epididymis in *Morn2*^+*/–*^ and *Morn2*^*–/–*^ mice. The flagellum mid-piece from *Morn2*^*–/–*^ spermatozoa showed hypertrophy and swelling of the mitochondria and disarrangement and hyperplasia of the outer dense fibers. Red arrow: outer dense fiber; White arrow: mitochondrial sheath. The test was replicated three times using distinct biological samples. The scale bar is shown in the figure. **B** SEM analysis of spermatozoa from the epididymis of *Morn2*^+*/–*^ and *Morn2*^*–/–*^ mice. The test was replicated three times using distinct biological samples. The scale bar is shown in the figure. **C** TEM showed that the spermatozoa from *Morn2*^*–/–*^ mice had axonemal disorganization. The test was replicated three times using distinct biological samples. The scale bar is shown in the figure. **D** TEM showed that the mid-piece of the axoneme from *Morn2*^*–/–*^ mice showed hyperplasia of the outer dense fiber, and the arrangement of the mitochondrial sheath was severely disrupted. Red arrow: outer dense fiber; Blue arrow: radial spoke; Yellow arrow: mitochondrial sheath; 1: microtubules doublet; 2: microtubule central pair. The test was replicated three times using distinct biological samples. The scale bar is shown in the figure

We next explored the sperm motility defects in *Morn2*^*–/–*^ mice using immunofluorescence analysis of the various motion-related structures in spermatozoa, but we found no obvious morphological abnormalities in the *Morn2*^*–/–*^ mice (Additional file 1: Fig. S3A-E). We then performed ultrastructural analysis with transmission electron microscopy (TEM) and scanning electron microscopy (SEM) to analyze the defects in *Morn2*^*–/–*^ spermatozoa in more detail. The TEM micrographs revealed that spermatozoa from *Morn2*^*–/–*^ mice showed hypertrophy and swelling of the mitochondria, which was accompanied by disarrangement and hyperplasia of the outer dense fibers in the flagellum mid-piece, while no obvious abnormalities were observed in the principal piece or end piece (Fig. 4A). The SEM analysis showed that the *Morn2*^*–/–*^ spermatozoa had different morphology compared to *Morn2*^+*/–*^, including bent heads, coiled flagella, mitochondrial sheath hypertrophy, and ruptured tails (Fig. 4B).

We used high-power TEM to examine longitudinal sections of the flagellum mid-piece, and we found that the mitochondria of *Morn2*^+*/–*^ spermatozoa were tightly packed around the axoneme with a regular arrangement and morphology, whereas the *Morn2*^*–/–*^ mitochondria had a disordered arrangement and failed to form a tight sheath around the axoneme (Fig. 4C). The mitochondria of *Morn2*^*–/–*^ mice were also variable in size and had anomalous morphologies (Fig. 4C). Analysis of flagellar mid-piece transverse sections showed that mitochondria of *Morn2*^+*/–*^ mice were fused into the expected tightly packed sheath structure, with the outer dense fibers arranged in neat rings inside the mitochondrial sheath (Fig. 4D). In contrast, *Morn2*^*–/–*^ mitochondria were not tightly packed, and the outer dense fibers presented as scattered structures with a radial distribution (Fig. 4D).

### *Morn2*^*–/–*^ sperm have abnormal energy metabolism

**Fig. 5:**
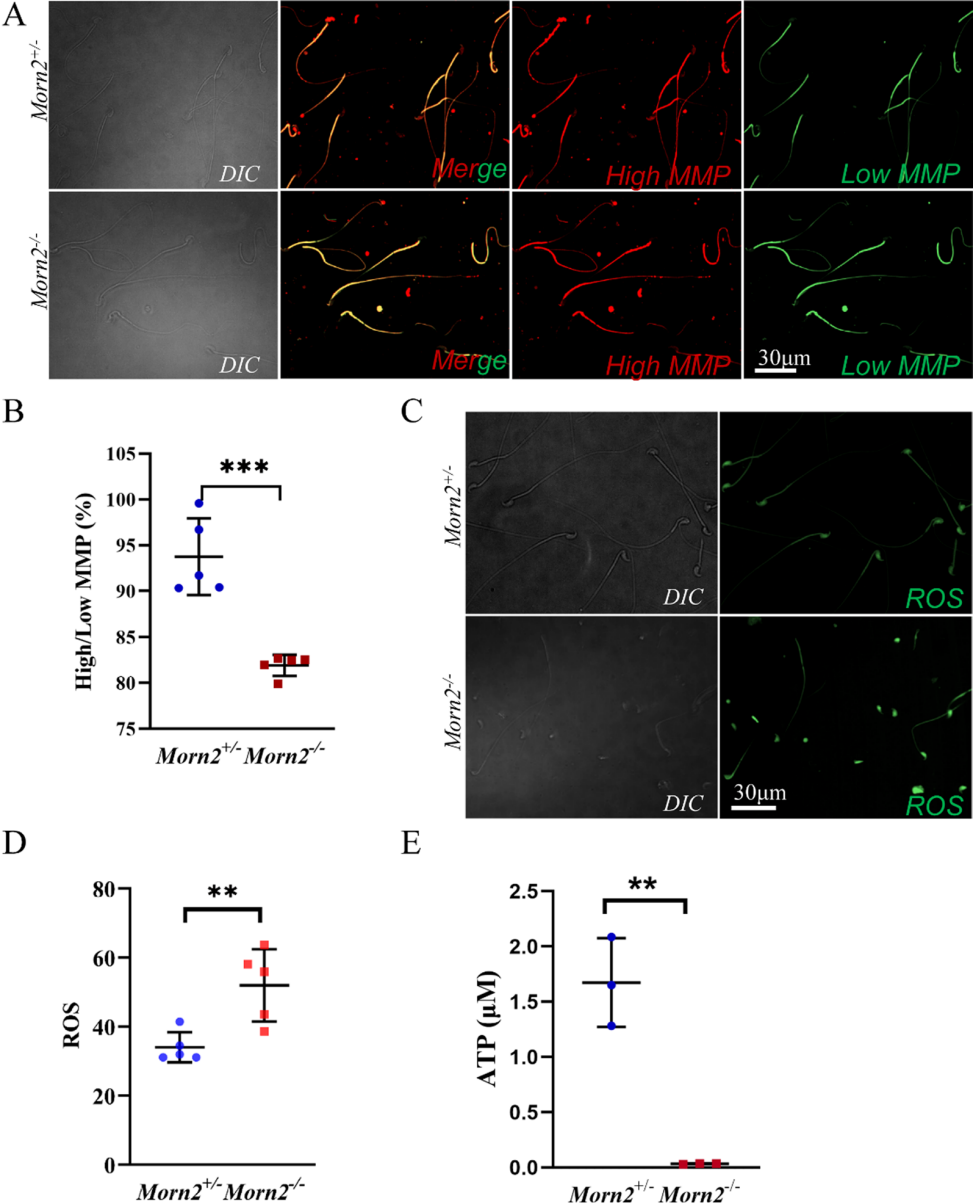
*Morn2*^*–/–*^ sperm show abnormal energy metabolism. **A** The MMP of spermatozoa collected from the cauda epididymis of adult *Morn2*^+*/–*^ and *Morn2*^*–/–*^ mice was measured using JC-1 as the marker. The morphology of sperm was examined with DIC. The test was replicated three times using distinct biological samples. The red fluorescent signal represents high MMP, while the green fluorescent signal represents low MMP. The scale bar is 30 μm. **B** The MMP levels of spermatozoa were indicated by the ratio of red to green fluorescence intensity signals of JC-1, with quantitative analysis of fluorescence signal intensity performed using ImageJ. The high/low MMP signal ratio in *Morn2*^*–/–*^ mice (81.93%) was lower than that in *Morn2*^+*/–*^ mice (93.75%). At least 200 spermatozoa were counted in each sample. ** = P < 0.01. The data are shown as the mean ± SEM of three independent experiments using distinct biological samples. Each data point represents the ratio of red to green fluorescence intensity signals of JC-1 in one sample, and Student’s t-tests were performed. **C** Analysis of ROS levels (green) in spermatozoa collected from the cauda epididymis of *Morn2*^+*/–*^ and *Morn2*^*–/–*^ adult mice. The morphology of sperm was examined with DIC. Fluorescence intensity represents the expression levels of ROS. The test was replicated three times using distinct biological samples. The scale bar is 30 μm. **D** The quantification of ROS fluorescence values was performed with the use of ImageJ. The ROS levels in *Morn2*^*–/–*^ mice (51.97) were higher than that in *Morn2*^+*/–*^ mice (34.01). At least 200 spermatozoa were counted in each sample. ** = P < 0.01. The data are shown as the mean ± SEM of three independent experiments using distinct biological samples. Each data point represents the ROS fluorescence intensity signals in one sample, and Student’s t-tests were performed. **E** Analysis of the ATP level in spermatozoa collected from the cauda epididymis of *Morn2*^+*/–*^ (1.673 μM) and *Morn2*^*–/–*^ (0.035 μM) adult mice. ** = P < 0.01. Data are shown as the mean ± SEM of three independent experiments using distinct biological samples. Each data point represents the ATP level of spermatozoa collected from one sample, and Student’s t-tests were performed

Decreased MMP is a manifestation of abnormal mitochondrial function and is a sign of early apoptosis [17]. To determine whether the abnormal morphology of mitochondria in *Morn2*^*–/–*^ spermatozoa affects the MMP, we used the fluorescent cationic carbocyanine dye JC-1, which labels mitochondria with high membrane potential red and low membrane potential green [22], as a probe to examine *Morn2*^+*/–*^ and *Morn2*^*–/–*^ sperm. We observed a reduced red/green signal ratio in the *Morn2*^*–/–*^ sperm suggesting their lower membrane potential and thus impaired mitochondrial function (Fig. 5A, B).

ROS are produced through the OXPHOS pathway and act as important secondary messengers that regulate intracellular pathways through oxidative activation. Excessive ROS production induces oxidative stress, thus causing spermatozoa damage and subsequent male infertility [12, 14]. We measured ROS levels in *Morn2*^+*/–*^ and *Morn2*^*–/–*^ spermatozoa using an ROS test kit (Fig. 5C) and found that the ROS level in *Morn2*^*–/–*^ spermatozoa was up-regulated (Fig. 5D, Additional file 1: Fig. S2G). These findings indicated that the balance between ROS and antioxidants was disrupted in *Morn2*^*–/–*^ mice thus inducing oxidative stress.

Mitochondria produce ATP through the OXPHOS pathway to power spermatozoa differentiation and motility [8], so we measured ATP levels in lysed *Morn2*^+*/–*^ and *Morn2*^*–/–*^ spermatozoa using an ATP test kit and a luminometer. The ATP concentration in *Morn2*^*–/–*^ spermatozoa was significantly reduced by an order of magnitude, suggesting that aerobic respiration in *Morn2*^*–/–*^ spermatozoa was severely impaired (Fig. 5E). Collectively, these results show that the knockout of *Morn2* causes lower MMP, higher ROS levels, and decreased ATP concentrations in spermatozoa, suggesting that *Morn2* is required for normal energy metabolism in spermatozoa.

## Discussion

The *Morn2* gene is evolutionarily conserved and testis-enriched, previous study predicted that MORN2 functions in the dynamic regulation of acrosome biogenesis during late spermiogenesis [18]. However, the subcellular localization of MORN2 and the specific regulatory pathways remain unclear. To explore the role of MORN2 in male fertility, we generated *Morn2*^*–/–*^ mice by deleting of exons 4–5. We found that *Morn2* regulates the morphology and energy metabolism of mitochondria and is essential for male fertility.

*Morn2*^*–/–*^ male mice were infertile, and *Morn2*^*–/–*^ sperm exhibited aberrant phenotypes. *Morn2*^*–/–*^ sperms were bent from the neck, their heads were attached to the flagellum, the proportion of deformed spermatozoa in *Morn2*^*–/–*^ was about 55.21 ± 6.32%. *Morn2*^*–/–*^ sperm also showed extremely decreased motility. Previous studies have reported that abnormal spermatozoa morphology can disturb sperm motility, and bending abnormalities have been linked to abnormal mitochondrial sheath morphology, although the mechanism underlying such bending has remained unclear [23]. These previous findings and our results presented above led us to hypothesize that *Morn2* plays a specific role in late spermatogenesis that affects the final morphology of spermatozoa.

The TEM and SEM micrographs revealed that spermatozoa from *Morn2*^*–/–*^ mice had defect in mitochondrial sheath formation. *Morn2*^*–/–*^ mitochondria were hypertrophy and swelling, they were variable in size and had anomalous morphologies. *Morn2*^*–/–*^ mitochondria also had a disordered arrangement and failed to form a tight sheath around the axoneme, suggesting that *Morn2* is required for the proper formation of the mitochondrial sheath. The ultrastructure and formation of the mitochondrial sheath have been described in previous studies, and knockout of several genes (*e.g.*, *ARMC12 *[3], *SPATA33 *[24], and *VDAC3 *[25, 26]) is known to cause abnormal mitochondrial sheath formation and subsequent infertility [27]. These studies have also shown that the integrated formation of the mitochondrial sheath is closely related to sperm motility [3, 25], thus these observations of morphological and structural abnormalities in the mitochondrial sheaths of *Morn2*^*–/–*^ spermatozoa are likely to contribute to the observed sperm motility defects and male infertility.

Although these specific factors (*e.g.*, ARMC12 [3], SPATA33 [24], VDAC3 [25, 26]) have been confirmed to be associated with abnormal mitochondrial sheath formation, studies targeting these molecules did not reveal mitochondrial metabolic abnormalities [3, 24, 25]. This suggests that normal mitochondrial sheath formation may not be necessary for energy metabolism in sperm.

*Morn2*^*–/–*^ sperm could successfully fertilize oocytes under the assistance of IVF. Thus, while some *Morn2*^*–/–*^ spermatozoa are competent for penetrating the ZP and fusing with oocytes, they could not complete fertilization in vivo, suggesting that the infertility of *Morn2*^*–/–*^ mice may be due to the inability of sperm to swim to oocytes at the appropriate time, rather than the dysfunction in sperm-egg fusion. We hypothesize that this is related to the poor motility of *Morn2*^*–/–*^ sperm, which are unable to transit through female reproductive tract and reach the ampulla in order to combine with the egg [28], thus resulting in male infertility. Given previous reports that the uterotubal junction (UTJ) can block the passage of sperm with some aberrant phenotypes [28, 29], whether the deletion of *MORN2* affects sperm passage through the UTJ remains to be assessed in further research. Collectively, our findings demonstrate that sperm from *Morn2*^*–/–*^ mice can penetrate the ZP and fuse with oocytes in vitro but are unable to complete fertilization in vivo, likely due to their poor motility thus resulting in male infertility.

Although our IVF results revealed that the *Morn2*^*–/–*^ sperm could complete in vitro fertilization, the fertilization rate was below normal (Fig. 3G). Previous studies have demonstrated the necessity of appropriate ROS concentrations in regulating the interaction between sperm and eggs, as well as fertilization. However, elevated ROS levels can induce cellular oxidative stress, leading to sperm DNA damage, subsequently affecting sperm-egg binding [12, 30], which may contribute to the observed reduction in fertilization rates of *Morn2*^*–/–*^ sperm.

MORN2 was initially identified as a protein that may be required for the dynamic regulation of acrosome biogenesis during late spermiogenesis [18], whereas in this study we found intact acrosomes in *Morn2*^*–/–*^ mice, suggesting that MORN2 is dispensable for acrosome biogenesis. *Morn2*^*–/–*^ mouse sperm exhibited extremely decreased ATP concentrations, but the detailed molecular mechanisms underlying this were not clear. ATP is a key product of the mitochondrial OXPHOS pathway [31, 32], and a previous study found that MORN2 is involved in LC3-associated phagocytosis (LAP) and promotes LAPosome maturation in macrophages in planarians [19]. A subsequent study reported that MORN2-mediated LAP is ROS-dependent and that MORN2 can influence ROS production by recruiting SNAP-23 onto phagosomes in macrophages [33]. The switch away from ATP production via OXPHOS to glycolytic metabolism in mitochondria is a hallmark of macrophages with pro-inflammatory phenotypes [34], and mitochondrial ROS generation is central to determining the inflammatory phenotype of macrophages, which repurpose mitochondria from ATP synthesis to ROS production [35]. We hypothesize that there is a similar mechanism in spermatozoa and that the knockout of MORN2 increases ROS production and/or induces a switch from ATP synthesis to ROS production, which would be aligned with the up-regulated ROS levels and significant decreases in ATP concentrations in *Morn2*^*–/–*^ mice sperm.

*Morn2* is an evolutionarily conserved and testis-enriched protein with two MORN motif domains [18]. Previous studies reported that via the MORN motif in its N-terminus, junctophilin type 2 (JP2) directly interacts with small-conductance Ca^2+^-activated K^+^ channel subtype2 (SK2) in mouse heart tissue [36] in order to modulate SK2 channel current amplitude and trafficking, thus regulating myocardial muscle contractions and Ca^2+^ homeostasis [20]. The MORN2 domain of JP2 was also found to enhance the membrane expression of SK2 channels and to increase *I*_k, Ca_ density [20]. Because SK2 is also expressed in testes [37], it was reasonable to speculate that MORN2 might interact with SK2 in the testes and thus affect electron trafficking and MMP in germ cells, thus regulating sperm motility.

Apoptosis is a programmed cell death process and a normal part of the cell lifecycle [38]. There are two main apoptotic signaling pathways: the extrinsic pathway of apoptosis and the intrinsic pathway, also known as the mitochondrial pathway [38]. Extensive research has confirmed that mitochondria are typically crucial for apoptosis [39]. Via mitochondrial outer membrane permeabilization (MOMP), the mitochondrial pathway releases soluble proteins from the mitochondrial intermembrane space, leading to cell death. In addition to inducing apoptosis, MOMP can also trigger pro-inflammatory signaling [38]. TUNEL staining of testicular sections showed that *Morn2*^*–/–*^ mice had an increased number of apoptotic spermatozoa, which may potentially be explained by mitochondrial dysfunction in *Morn2*^*–/–*^ mice.

Recently, the high rates of unintended pregnancies have arisen a pressing need to broaden contraceptive choices. The research on novel male contraceptive methods has also garnered wide attention. Non-hormonal male contraceptive approaches, primarily focusing on disrupting sperm motility or sperm-egg binding, have become a major research focus [40]. The knockout of *Morn2* can lead to decreased sperm motility and a decline in fertilization rates, suggesting that *Morn2* may emerge as a novel target in male contraceptive research.

## Conclusion

Our present findings demonstrated for the first time that *Morn2* may not only participate in the formation of mitochondrial sheath, but may also be required for energy metabolism by sperm mitochondria. Thus, our study provides insights about a potential causal gene for asthenospermia, and identifies a potential target for the development of male contraception technologies.

### Supplementary Information

**Additional file 1: Figure S1.A** RT-PCR analysis of the indicated mRNAs in wild type adult mouse tissues with Gapdh serving as the reference control. The test was replicated three times using distinct biological samples.** B** RT-PCR analysis with the indicated mRNAs of Morn2 from testes at different ages with Gapdh serving as the reference control. The test was replicated three times using distinct biological samples.** Figure S2.** **A** Immunofluorescence co-staining for the acrosomal marker PNA (red) and the nuclear marker DAPI (blue) in *Morn2*^*+/–*^ and *Morn2*^*–/–*^ spermatozoa of adult mice indicating that acrosome development was intact in *Morn2*^*–/–*^ male mice. The test was replicated three times using distinct biological samples. The scale bar is 2 μm. **B** Immunofluorescence co-staining for the Golgi marker GM130 (red), the acrosome marker lectin (green), and the nuclear marker DAPI (blue) in the testes of adult *Morn2*^*+/–*^ and *Morn2*^*–/–*^ mice. The test was replicated three times using distinct biological samples. The scale bar is 30 μm.** C** Immunofluorescence co-staining for the Golgi marker GOPC (red) and the nuclear marker DAPI (blue) in the testes of adult *Morn2*^*+/–*^ and *Morn2*^*–/–*^ mice. The test was replicated three times using distinct biological samples. The scale bar is 30 μm.** D** The percentage of epididymal sperm with bent flagella in *Morn2*^*+/–*^ (6.30 ± 0.90 %) and *Morn2*^*–/–*^ (7.68 ± 1.64 %) mice. The data are shown as the mean ± SEM of three independent experiments using distinct biological samples. Each data point represents the percentage of sperm with bent flagella per sample, ns = P > 0.05 by Student’s t-test.** E** The percentage of epididymal sperm with coiled tails in *Morn2*^*+/–*^ (1.23 ± 0.68 %) and *Morn2*^*–/–*^ (2.95 ± 1.95 %) mice. The data are shown as the mean ± SEM of three independent experiments using distinct biological samples. Each data point represents the percentage of sperm with coiled tails per sample, ns = P > 0.05 by Student’s t-test. **F** In *Morn2*^*–/–*^ mice, the percentage of sperm with abnormal phenotype in the caput, corpora, and cauda epididymidies was 64.34% (caput), 61.30% (corpora), and 55.21% (cauda), and in *Morn2*^*+/+*^ mice the percentage of sperm with abnormal phenotype was 29.71% (caput), 27.98% (corpora), and 11.28% (cauda), the quantity of abnormal sperm in *Morn2*^*–/–*^ mice was significantly higher than that in the *Morn2*^*+/+*^ mice of the corresponding period. At least 200 spermatozoa were counted in each sample. The data are shown as the mean ± SEM of three independent experiments using distinct biological samples. **** = P < 0.0001, *** = P < 0.001 by Student’s t-tests. **G **The quantification of ROS fluorescence values was performed with the use of fluorescence luminometer. The ROS levels in *Morn2*^*–/–*^ mice (1.18) were higher than that in *Morn2*^*+/–*^ mice (1.00). At least 200 spermatozoa were counted in each sample. ** = P < 0.01. The data are shown as the mean ± SEM of three independent experiments using distinct biological samples, each data point represents the ROS fluorescence value of one sample, and Student’s t-tests were performed.** Figure S3. A** Immunofluorescence co-staining of the mitochondrial marker Mito tracker (red) and DAPI (blue) in *Morn2*^*+/–*^ and *Morn2*^*–/–*^ spermatozoa of adult mice. The test was replicated three times using distinct biological samples. The scale bar is 15 μm.** B** Immunofluorescence co-staining of the mitochondrial marker TOMM 20 (red) and DAPI (blue) in *Morn2*^*+/–*^ and *Morn2*^*–/–*^ spermatozoa of adult mice. The morphology of sperm was examined with DIC. The test was replicated three times using distinct biological samples. The scale bar is 15 μm.** C** Immunofluorescence co-staining of the inner dynein arm marker DNALI1 (green) and DAPI (blue) in *Morn2*^*+/–*^ and *Morn2*^*–/–*^ spermatozoa of adult mice. The morphology of sperm was examined with DIC. The test was replicated three times using distinct biological samples. The scale bar is 10 μm.** D** Immunofluorescence co-staining of the central microtubule marker SPEF2 (green) and DAPI (blue) in *Morn2*^*+/–*^ and *Morn2*^*–/–*^ spermatozoa of adult mice. The morphology of sperm was examined with DIC. The test was replicated three times using distinct biological samples. The scale bar is 15 μm. **E** Immunofluorescence co-staining of the flagellum marker SPAG6 (green) and DAPI (blue) in *Morn2*^*+/–*^ and *Morn2*^*–/–*^ spermatozoa of adult mice. The morphology of sperm was examined with DIC. The test was replicated three times using distinct biological samples. The scale bar is15 μm.**Table S1.** Antibody Information

## Data Availability

The datasets generated and/or analyzed during the present study are available from the corresponding author on reasonable request.
