# Supplementary material for: MORN2 regulates the morphology and energy metabolism of mitochondria and is required for male fertility in mice
Source: J Transl Med. 2024 Mar 5;22:240. doi: 10.1186/s12967-024-05010-3 (PMC10916217; doi:10.1186/s12967-024-05010-3)
Supplement: Supplementary file 1 — Additional file 1: Figure S1.A RT-PCR analysis of the indicated mRNAs in wild type adult mouse tissues with Gapdh serving as the reference control. The test was replicated three times using distinct biological samples. B RT-PCR analysis with the indicated mRNAs of Morn2 from testes at different ages with Gapdh serving as the reference control. The test was replicated three times using distinct biological samples. Figure S2. A Immunofluorescence co-staining for the acrosomal marker PNA (red) and the nuclear marker DAPI (blue) in Morn2+/– and Morn2–/– spermatozoa of adult mice indicating that acrosome development was intact in Morn2–/– male mice. The test was replicated three times using distinct biological samples. The scale bar is 2 μm. B Immunofluorescence co-staining for the Golgi marker GM130 (red), the acrosome marker lectin (green), and the nuclear marker DAPI (blue) in the testes of adult Morn2+/– and Morn2–/– mice. The test was replicated three times using distinct biological samples. The scale bar is 30 μm. C Immunofluorescence co-staining for the Golgi marker GOPC (red) and the nuclear marker DAPI (blue) in the testes of adult Morn2+/– and Morn2–/– mice. The test was replicated three times using distinct biological samples. The scale bar is 30 μm. D The percentage of epididymal sperm with bent flagella in Morn2+/– (6.30 ± 0.90 %) and Morn2–/– (7.68 ± 1.64 %) mice. The data are shown as the mean ± SEM of three independent experiments using distinct biological samples. Each data point represents the percentage of sperm with bent flagella per sample, ns = P > 0.05 by Student’s t-test. E The percentage of epididymal sperm with coiled tails in Morn2+/– (1.23 ± 0.68 %) and Morn2–/– (2.95 ± 1.95 %) mice. The data are shown as the mean ± SEM of three independent experiments using distinct biological samples. Each data point represents the percentage of sperm with coiled tails per sample, ns = P > 0.05 by Student’s t-test. F In Morn2–/– mice, the percentage of sperm [file 12967_2024_5010_MOESM1_ESM.zip › Supplementary/Supplementary figures and captions.docx]

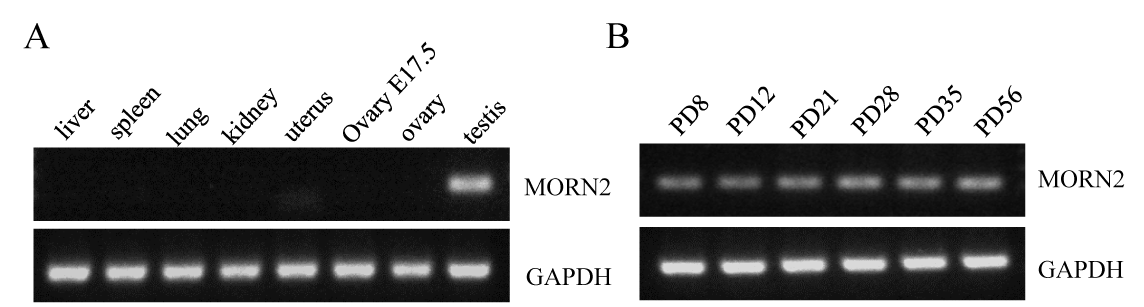


**Figure S1:**

(A) RT-PCR analysis of the indicated mRNAs in wild type adult mouse tissues with *Gapdh* serving as the reference control. The test was replicated three times using distinct biological samples.

(B) RT-PCR analysis with the indicated mRNAs of *Morn2* from testes at different ages with *Gapdh* serving as the reference control. The test was replicated three times using distinct biological samples.


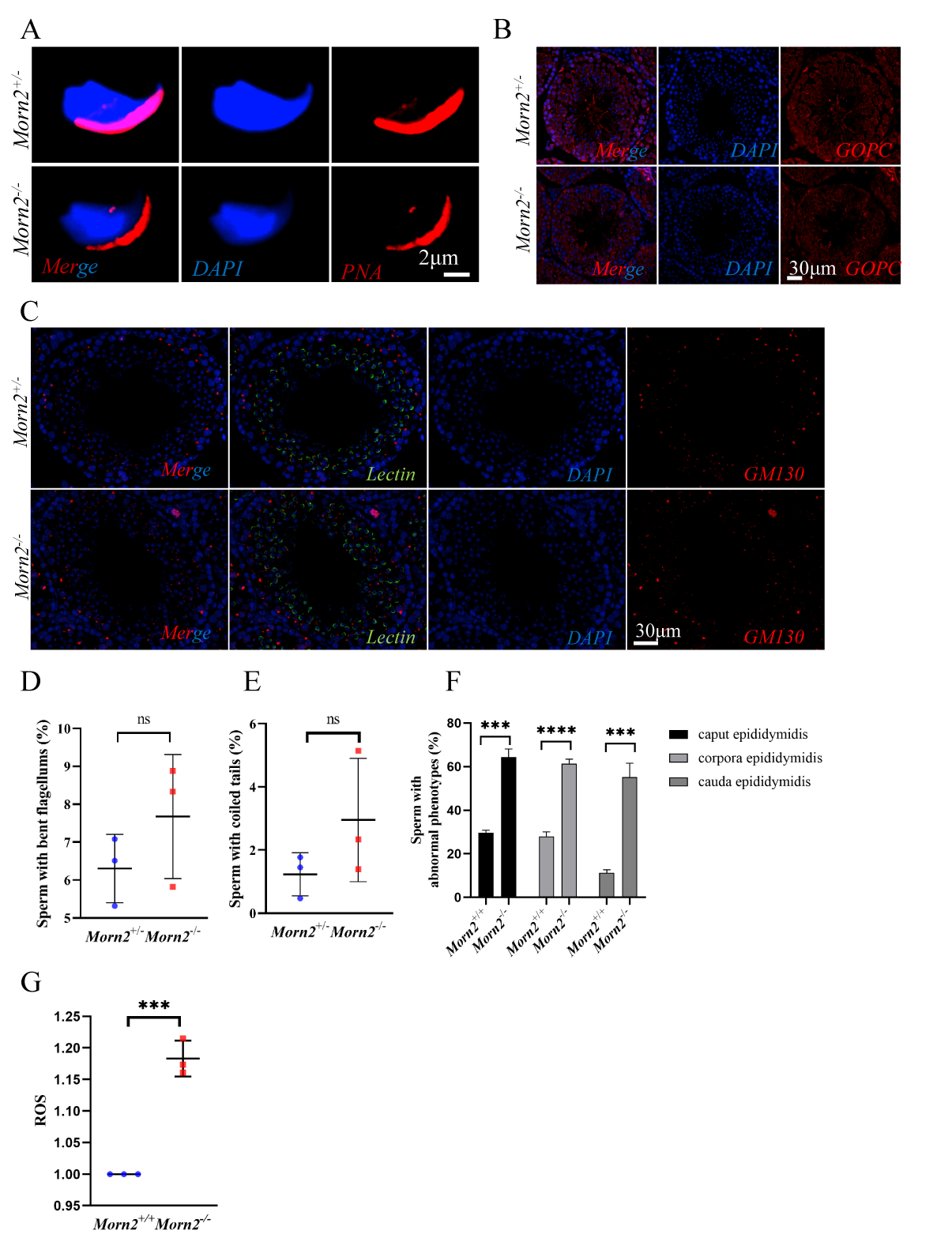


**Figure S2:**

1. Immunofluorescence co-staining for the acrosomal marker PNA (red) and the nuclear marker DAPI (blue) in *Morn2^+/–^* and *Morn2*^–/–^ spermatozoa of adult mice indicating that acrosome development was intact in *Morn2*^–/–^ male mice. The test was replicated three times using distinct biological samples. The scale bar is 2 μm.
2. Immunofluorescence co-staining for the Golgi marker GM130 (red), the acrosome marker lectin (green), and the nuclear marker DAPI (blue) in the testes of adult *Morn2^+/–^* and *Morn2*^–/–^ mice. The test was replicated three times using distinct biological samples. The scale bar is 30 μm.
3. Immunofluorescence co-staining for the Golgi marker GOPC (red) and the nuclear marker DAPI (blue) in the testes of adult *Morn2^+/–^* and *Morn2*^–/–^ mice. The test was replicated three times using distinct biological samples. The scale bar is 30 μm.
4. The percentage of epididymal sperm with bent flagella in *Morn2^+/–^* (6.30 ± 0.90 %) and *Morn2^–/–^* (7.68 ± 1.64 %) mice. The data are shown as the mean ± SEM of three independent experiments using distinct biological samples. Each data point represents the percentage of sperm with bent flagella per sample, ns = P > 0.05 by Student’s t-test.
5. The percentage of epididymal sperm with coiled tails in *Morn2^+/–^* (1.23 ± 0.68 %) and *Morn2^–/–^* (2.95 ± 1.95 %) mice. The data are shown as the mean ± SEM of three independent experiments using distinct biological samples. Each data point represents the percentage of sperm with coiled tails per sample, ns = P > 0.05 by Student’s t-test.
6. In *Morn2^–/–^* mice, the percentage of sperm with abnormal phenotype in the caput, corpora, and cauda epididymidies was 64.34% (caput), 61.30% (corpora), and 55.21% (cauda), and in *Morn2^+/+^* mice the percentage of sperm with abnormal phenotype was 29.71% (caput), 27.98% (corpora), and 11.28% (cauda), the quantity of abnormal sperm in *Morn2^–/–^* mice was significantly higher than that in the *Morn2^+/+^* mice of the corresponding period. At least 200 spermatozoa were counted in each sample. The data are shown as the mean ± SEM of three independent experiments using distinct biological samples. **** = P < 0.0001, *** = P < 0.001 by Student’s t-tests.
7. The quantification of ROS fluorescence values was performed with the use of fluorescence luminometer. The ROS levels in *Morn2^–/–^* mice (1.18) were higher than that in *Morn2^+/–^* mice (1.00). At least 200 spermatozoa were counted in each sample. ** = P < 0.01. The data are shown as the mean ± SEM of three independent experiments using distinct biological samples, each data point represents the ROS fluorescence value of one sample, and Student’s t-tests were performed.


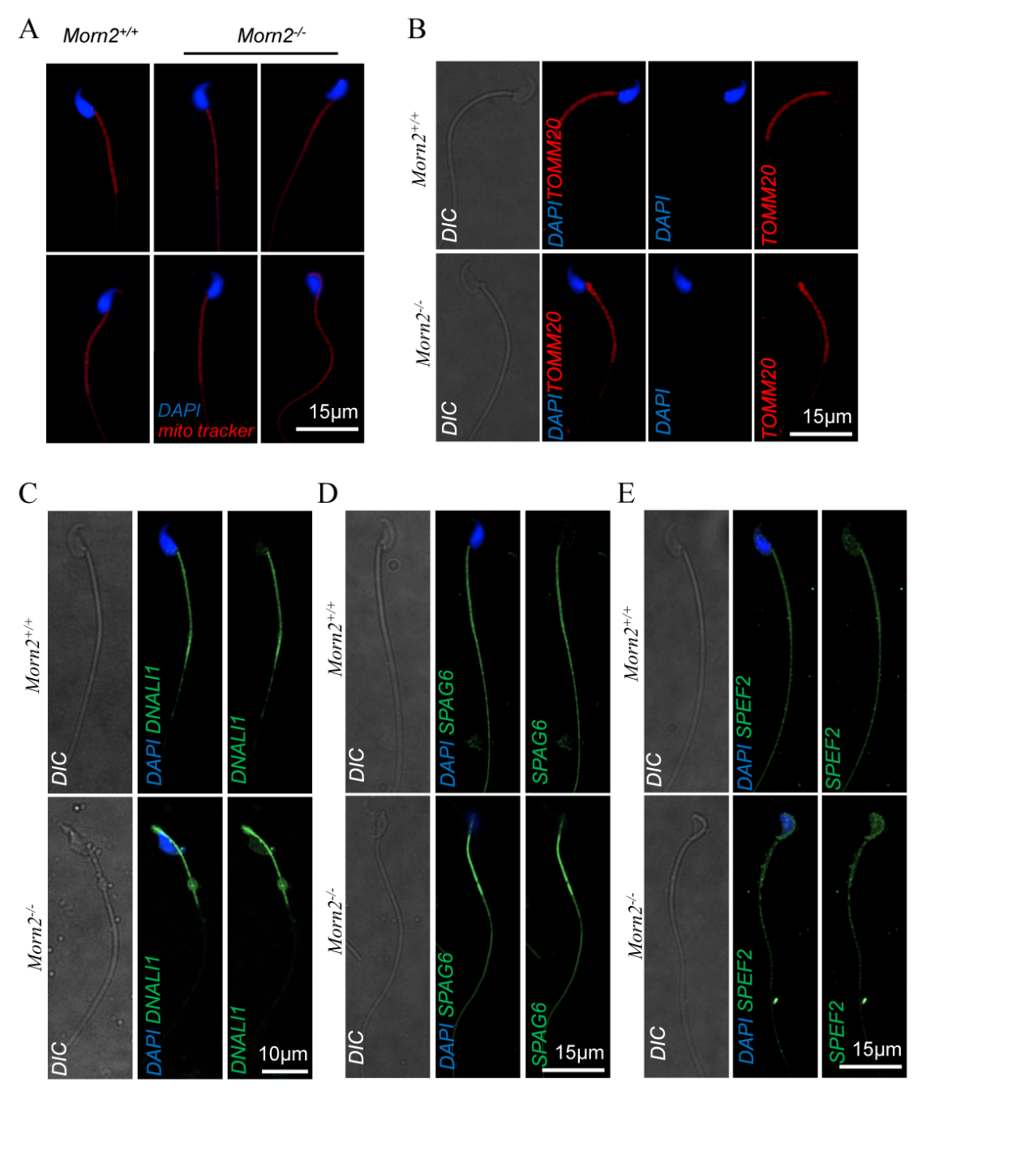


**Figure S3:**

1. Immunofluorescence co-staining of the mitochondrial marker Mito tracker (red) and DAPI (blue) in *Morn2^+/–^* and *Morn2*^–/–^ spermatozoa of adult mice. The test was replicated three times using distinct biological samples. The scale bar is 15 μm.
2. Immunofluorescence co-staining of the mitochondrial marker TOMM 20 (red) and DAPI (blue) in *Morn2^+/–^* and *Morn2*^–/–^ spermatozoa of adult mice. The morphology of sperm was examined with DIC. The test was replicated three times using distinct biological samples. The scale bar is 15 μm.
3. Immunofluorescence co-staining of the inner dynein arm marker DNALI1 (green) and DAPI (blue) in *Morn2^+/–^* and *Morn2*^–/–^ spermatozoa of adult mice. The morphology of sperm was examined with DIC. The test was replicated three times using distinct biological samples. The scale bar is 10 μm.
4. Immunofluorescence co-staining of the central microtubule marker SPEF2 (green) and DAPI (blue) in *Morn2^+/–^* and *Morn2*^–/–^ spermatozoa of adult mice. The morphology of sperm was examined with DIC. The test was replicated three times using distinct biological samples. The scale bar is 15 μm.
5. Immunofluorescence co-staining of the flagellum marker SPAG6 (green) and DAPI (blue) in *Morn2^+/–^* and *Morn2*^–/–^ spermatozoa of adult mice. The morphology of sperm was examined with DIC. The test was replicated three times using distinct biological samples. The scale bar is15 μm.
