# Supplementary material for: MORN2 regulates the morphology and energy metabolism of mitochondria and is required for male fertility in mice
Source: J Transl Med. 2024 Mar 5;22:240. doi: 10.1186/s12967-024-05010-3 (PMC10916217; doi:10.1186/s12967-024-05010-3)
Supplement: Supplementary file 1 — Additional file 1: Figure S1.A RT-PCR analysis of the indicated mRNAs in wild type adult mouse tissues with Gapdh serving as the reference control. The test was replicated three times using distinct biological samples. B RT-PCR analysis with the indicated mRNAs of Morn2 from testes at different ages with Gapdh serving as the reference control. The test was replicated three times using distinct biological samples. Figure S2. A Immunofluorescence co-staining for the acrosomal marker PNA (red) and the nuclear marker DAPI (blue) in Morn2+/– and Morn2–/– spermatozoa of adult mice indicating that acrosome development was intact in Morn2–/– male mice. The test was replicated three times using distinct biological samples. The scale bar is 2 μm. B Immunofluorescence co-staining for the Golgi marker GM130 (red), the acrosome marker lectin (green), and the nuclear marker DAPI (blue) in the testes of adult Morn2+/– and Morn2–/– mice. The test was replicated three times using distinct biological samples. The scale bar is 30 μm. C Immunofluorescence co-staining for the Golgi marker GOPC (red) and the nuclear marker DAPI (blue) in the testes of adult Morn2+/– and Morn2–/– mice. The test was replicated three times using distinct biological samples. The scale bar is 30 μm. D The percentage of epididymal sperm with bent flagella in Morn2+/– (6.30 ± 0.90 %) and Morn2–/– (7.68 ± 1.64 %) mice. The data are shown as the mean ± SEM of three independent experiments using distinct biological samples. Each data point represents the percentage of sperm with bent flagella per sample, ns = P > 0.05 by Student’s t-test. E The percentage of epididymal sperm with coiled tails in Morn2+/– (1.23 ± 0.68 %) and Morn2–/– (2.95 ± 1.95 %) mice. The data are shown as the mean ± SEM of three independent experiments using distinct biological samples. Each data point represents the percentage of sperm with coiled tails per sample, ns = P > 0.05 by Student’s t-test. F In Morn2–/– mice, the percentage of sperm [file 12967_2024_5010_MOESM1_ESM.zip › Supplementary/Table S1.docx]

Table S1: Antibody Information

| **Protein** | **Manufacture (catalogue number)** | **Applications (working dilution)** |
| --- | --- | --- |
| GM130 | BD (610822) | IF (1:200) |
| GOPC | Abcam (ab37036) | IF (1:200) |
| Lectin (PNA) | Invitrogen(L32459) | IF (1:1000) |
| Lectin (PNA) | Invitrogen (L21409) | IF (1:1000) |
| Mito Tracker | Invitrogen (M22426) | IF (1:1000) |
| TOMM20 | Abcam (ab221292) | IF (1:500) |
| DNALI1 | Sigma (HPA028305) | IF (1:200) |
| SPAG6 | Sigma (HPA038440) | IF (1:200) |
| SPEF2 | Sigma (HPA040343) | IF (1:200) |
| Alexa Fluor 488 Goat anti-Mouse IgG | Abcam (ab150117) | IF (1:500) |
| Alexa Fluor 488 Goat anti-Rabbit IgG | Abcam (ab150077) | IF (1:500) |
| Alexa Fluor 594 Goat anti-Mouse IgG | Abcam (ab150120) | IF (1:500) |
| Alexa Fluor 594 Goat anti-Rabbit IgG | Abcam (ab150080) | IF (1:500) |
